# Morphometric Assessment of Temporomandibular Joint Space in Dentate and Edentulous Patients by Using Cone Beam Computed Tomography

**DOI:** 10.7759/cureus.69692

**Published:** 2024-09-19

**Authors:** Hilal Demir, Bilgun Cetin

**Affiliations:** 1 Department of Oral and Maxillofacial Radiology, Beyhekim Oral and Dental Health Center, Konya, TUR; 2 Department of Oral and Maxillofacial Radiology, Selçuk University, Faculty of Dentistry, Konya, TUR

**Keywords:** cbct, cone‐beam computed tomography, glenoid fossa, mandibular condyle, temporomandibular joint spaces, tmj disorder

## Abstract

Aim: This study aimed to determine the changes in the temporomandibular joint due to ethnicity and to reveal gender differences.

Methods: This retrospective study included the evaluation of cone beam computed tomography (CBCT) images of 110 patients (220 temporomandibular joint {TMJs}), 64 dentulous (32 females, 32 males) and 46 edentulous (18 females, 28 males). Anterior, superior, and posterior joint spaces and the roof of the glenoid fossa (GF) were measured in sagittal views. Shapiro-Wilk test, independent sample t-test, and Mann-Whitney U tests were used to determine significant differences between dentulous and edentulous patients.

Results: The difference in terms of anterior and posterior joint space was statistically significant (p=0.005, p˂0.001). However, no statistically significant difference was found in the upper joint space (p=0.227). A statistically significant difference was not found in GF roof thickness (p=0.229).

Conclusions: Upper joint space and GF roof thickness were not associated with edentulism. However, this situation is directly related to both posterior and anterior joint spaces, and this relationship manifests itself as a decrease in the posterior joint space and an increase in the anterior joint space with edentulism.

## Introduction

The temporomandibular joint (TMJ) is a multidirectional synovial joint that attaches the anatomical structures of the gnathologic system, the mandibular condyle, the glenoid fossa (GF), and the articular process of the temporal bone [1]. The TMJ consists of soft tissue constituents, such as the articular disc, joint capsule, articular ligaments, and retrodiscal tissue, and bony components, such as mandibular condyle, GF, and articular eminence. The GF, also known as the mandibular fossa, is the concave area positioned in the inferior region of the squamous portion of the temporal bone where the condyle is located. The part of the GF that is functional and covered with articular tissue is called the articular fossa [2]. The radiographic joint space is defined as the area of decreased radiopacity situated between the outer cortical layer of the temporal bone and the outer cortical layer of the mandibular condyle. Adequate joint space is essential for facilitating condyle movement with the articular disc. Temporal changes in the position of the condyle can cause alterations in the TMJ surfaces, resulting in signs of temporomandibular joint disorders, including dysfunction and discomfort [1]. TMJs are one of the joints with the most movement dynamics in the human body, undergoing over 2000 daily movements during activities, such as biting, chewing, swallowing, and speaking [3].

The global population of individuals aged 65 years and above is experiencing a growing trend. The estimated rate in 2015 was 8.5%, and it is projected to rise to 12% in 2030 and 16.7% in 2050. The exponential growth of the older adult population has transformed toothless into a pressing public health concern that demands immediate attention and proactive intervention. Full toothlessness means the complete lack of natural teeth or roots in the entire dental arch. The stomatognathic system experiences destabilization in the absence of an occlusal relationship with toothless patients [4].

The force and movement of the TMJ are directly influenced by tooth contact. The TMJ frequently experiences structural remodeling and degenerative changes as it adjusts to alterations in occlusal function. Pathological degenerative changes manifest when the structural alterations of the TMJ exceed the physiological thresholds of the human body. Consequently, alterations in the stress conditions of the TMJ during jaw movements in patients without teeth (edentulous patients) can cause modifications in the anatomical structure and result in functional disorders [4,5].

In terms of the effect of teeth lack on TMJ spaces, research has demonstrated that the superior and posterior substitution of the condyles within the joint space can result from tooth wear and a reduction in vertical face height caused by tooth loss [1]. Amorim et al. determined a substantial decrease in posterior joint space as a result of the loss of posterior teeth [6]. Similarly, Yanıkoğlu and Guldag conducted a study that indicated that individuals with bilateral lack of posterior teeth support zones displayed a diminution in posterior, superior, and anterior joint spaces, in contrast to those with unilateral lack of posterior teeth support zones [5]. Tabatabaei et al. demonstrated that posterior edentulism resulted in a reduction in the posterior and upper joint spaces, while the anterior joint space did not exhibit any significant changes [1]. Arıkan et al. compared the anterior, upper, and posterior joint spaces of edentulous patients to those of dentate patients, and they did not observe any statistically significant variations in any of the measured joint spaces, despite the fact that changes in TMJ joint spaces had been observed in previous studies [7].

The roof of the GF is a thin bone that separates the TMJ from the middle cranial fossa [8]. The observation that the roof is thinner compared to other areas of the GF suggests that this structure may not be well-suited to endure extreme forces. It has been documented that a reduction in roof thickness is linked to perforation of the articular disc and that osteoarthritic changes can impact the thickness of the GF roof due to mechanical stress and force distribution. Additionally, as patients age increases, their capacity for tissue repair may be diminished [9]. Finally, Rosado et al. showed a decrease in GF roof thickness in edentulous patients compared to edentulous patients [9].

Different imaging techniques, such as conventional radiography, cone beam computed tomography (CBCT), medical computed tomography (CT), ultrasound, and magnetic resonance imaging (MRI) can be utilized to study the complex structure of the TMJ [3,10]. The use of CBCT has become an increasingly important resource in diagnosing TMJ disorder, particularly in evaluating structural changes in the mandibular condyles [11]. CBCT is a cost-effective and relatively low-dose imaging modality that offers high-resolution images for the diagnostic assessment of the bone structures of the TMJ [12,13].

Unlike the previous studies that have assessed either the joint space or the glenoid fossa floor separately, this study aimed to evaluate both aspects within the same individuals using CBCT [1,4,5,8,9]. Therefore, it aimed to determine the change of edentulism in the joint and reveal gender differences.

## Materials and methods

The Non-interventional Clinical Applications Ethics Committee of the Selçuk University, Faculty of Dentistry issues approval for the current study under the number 2024/36. Power analysis was performed (by G*power version 3.1) to determine the number of individuals to be included in the study. Sample calculation was performed for an independent sample t-test. According to the data obtained from the reference study, the effect size was calculated as 0.6345021 [9]. Accordingly, with an effect size of 0.6345021 (d=0.6345021), 5% margin of error (α=0.05), and 90% power (1-β=0.90), the sample size for each group was calculated as 44. According to these data, the number of individuals in each group was planned to be at least 44, and the total sample size was planned to be 88.

CBCT images were selected by scanning the image archive of the Department of Oral and Maxillofacial Radiology, Faculty of Dentistry, Selçuk University, between 2022 and 2024. Out of a total of 1164 CBCT images, 64 images of patients with complete dentition and 46 images of patients with complete edentulous teeth were selected for evaluation. The measurements were performed on CBCT images of 110 patients (220 TMJs), 64 with teeth (32 females, 32 males) and 46 without teeth (18 females, 28 males). The individuals’ ages ranged from 20 to 75 years. The edentulous patients in the study were completely edentulous, while the control group consisted of patients with at least one tooth in each quadrant that provided occlusal contact without changing the vertical dimension. Patients with severe skeletal malocclusion, developmental congenital anomalies, or any systemic conditions that could induce morphological alterations in the joint, like rheumatoid arthritis, were not included in the research. Additionally, individuals with acute trauma, a history of temporomandibular surgery, musculoskeletal or neurological disorders, and those with cysts or tumors on the TMJ bone surface were not included. Additionally, patients were excluded from the study if their CBCT images had an insufficient field of view (FOV) that did not capture both TMJ regions or if the images were of inadequate quality due to artifacts.

CBCT of TMJs

Selected CBCT images of bilateral TMJs were obtained using an Instrumentarium Dental device (Tuusula, Finland: PaloDEx Group) with a 13 × 15 cm field of view. The exposure factors used during the scans were set to 90 kV, 5 mA, and 8.1 s exposure time. Subjects stood while biting their teeth in the maximal intercuspation position, with their heads adjusted so that the Frankfurt plane was positioned parallel to the floor. The images were evaluated using a high-resolution Asus Desktop-47CM0VT monitor in a customized reporting room located in a dimly lit environment with appropriate viewing conditions.

**Figure 1:**
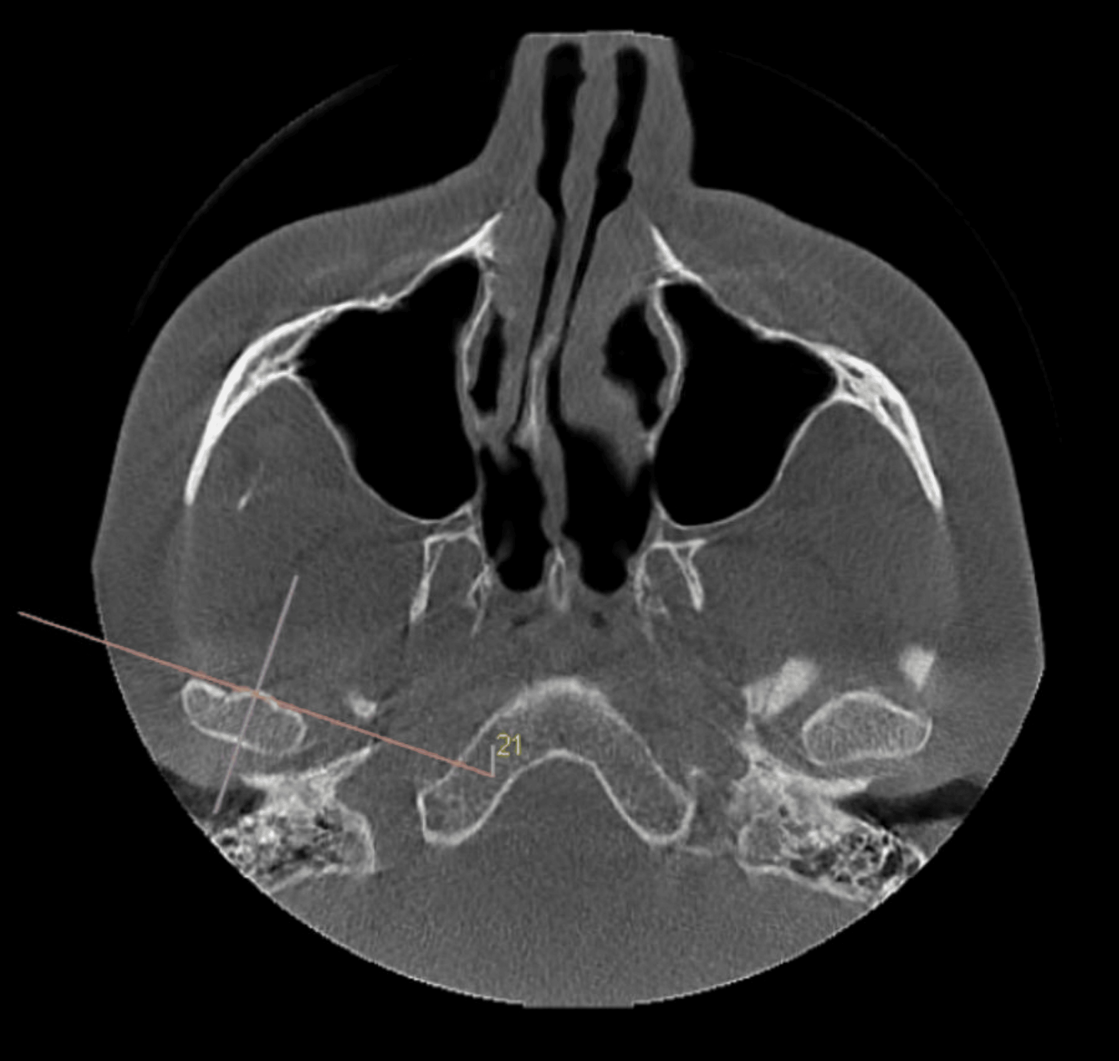
Axial slice of cone beam computed tomography (CBCT) image.

**Figure 2:**
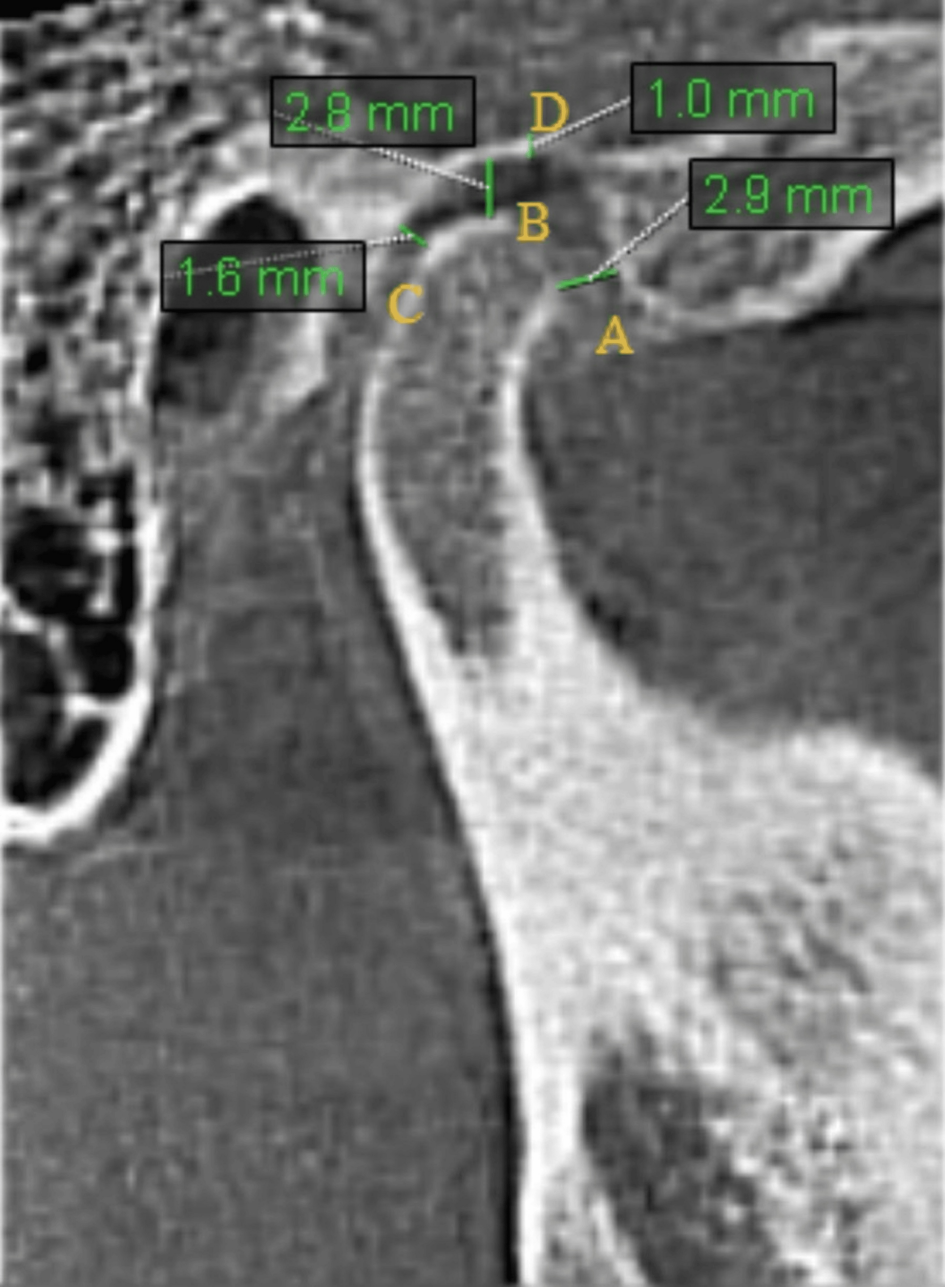
Reconstructed sagittal slice of cone beam computed tomography (CBCT) image. (A) Anterior joint space, (B) superior joint space, (C) posterior joint space, (D) glenoid fossa roof thickness

For image analysis, the greatest mediolateral diameter of the condylar process axial view within the TMJ window was selected (Figure 1). In this image, a line running parallel to the longitudinal axis of the condylar process was created, and the sagittal slices were reconstructed to form 0.5 mm thick slices. In the central sagittal slice, the narrowest posterior, anterior, and superior joint spaces on the left and right sides and the roof thickness of the glenoid fossa were measured (Figure 2). All CBCT images were evaluated by a radiologist with seven years of experience, and measurements were repeated two weeks after the initial measurement to assess the reliability of the observer’s measurement.

Statistical analyses

IBM SPSS version 22 (Armonk, NY: IBM Corp.) was used to analyze the data in this study. The agreement between the measurements made by the observer was determined using the intraclass correlation coefficient (ICC). The normality of the measurement distribution was evaluated using the Shapiro-Wilk test. Group differences in normally distributed data were analyzed with the independent sample t-test, while non-normally distributed data were assessed using the non-parametric Mann-Whitney U test. The p-value’s significance level was determined to be 0.05.

## Results

The measurements of the GF roof thickness had an intraclass correlation coefficient (ICC) of 0.890, while the anterior, superior, and posterior joint spaces had ICCs of 0.906, 0.947, and 0.845, respectively. After the ICC displayed nearly flawless agreement, additional analysis was conducted using the second measurement.

The ages of the edentulous patients were 35-75 (61.28±9.78) years, while the ages of the dentulous patients were 20-55 (35.44±10.87) years. Fifty (45.4%) of the 110 patients were female, and 60 (55.6%) were male. Among the dentulous patients, 32 (50%) were female, and 32 (50%) were male. Among the edentulous patients, 18 (39%) were female and 28 (61%) were male (Table 1).

**Table 1:** Age and gender distribution of full dentate and edentulous patients. N: number

Variables	Edentulous		Dentate		Total
	Females	Males	Females	Males	
N (%)	18 (39%)	28 (61%)	35 (50%)	35 (50%)	110 (100%)
Age	59.66±14.12	62.88±7.37	35.21±9.88	39.22±14.71	49.26±16.59
Total	61.28±9.78		35.44±10.87		-

The mean anterior joint space was 3.07 mm in the edentulous patients and 2.62 mm in the dentulous patients. The mean superior joint space was 3.95 mm in the edentulous patients and 3.56 mm in the dentulous patients. The mean posterior joint space was 1.62 mm in the edentulous patients and 1.95 mm in the dentulous patients. The difference in the anterior and posterior joint space was statistically significant (p=0.005, p<0.001). However, no statistically significant difference was obtained in the superior joint space (p=0.227). The mean GF roof thickness was 1.87 mm in the edentulous patients and 1.77 mm in the dentulous patients. Statistically, no significant difference was obtained in GF roof thickness (p=0.229) (Table 2).

**Table 2:** Mean±standard deviation and p-values of ant., sup., and post. joint spaces and GF roof thickness in relation to edentulous and full dentate patients. *Statistically significant values. N: number; GF: glenoid fossa; Ant.: anterior; Sup.: superior; post.: posterior

Joint space and GF	Edentulous	Dentate	p-Value
Ant.	3.07±1.21 (mm)	2.62±0.87 (mm)	0.005*
Sup.	3.95±1.15 (mm)	3.56±1.03 (mm)	0.227
Post.	1.62±0.54 (mm)	1.95±0.57 (mm)	<0.001*
GF	1.87±0.65 (mm)	1.77±0.64 (mm)	0.229
N	128	92	-

Tables 3, 4 present a comparison of the measurement mean values by dentate status and sex. A significant difference was found between genders in dentulous patients for anterior joint space and roof thickness (p=0.023, p=0.005). In edentulous patients, anterior, superior, and posterior joint spaces were statistically significant between males and females (p=0.012, p=0.019, and p=0.007).

**Table 3:** Mean±standard deviation and p-values of ant., sup., and post. joint spaces (mm) and GF roof thickness (mm) in relation to genders of full dentate patients. *Statistically significant values. N: number; GF: glenoid fossa; Ant.: anterior; Sup.: superior; post.: posterior

Joint space and GF thickness measurements	Females		Males		p-Value
	Measurements (mm)	N	Measurements (mm)	N	
Ant.	2.40±0.72	64	2.79±0.98	64	0.023*
Sup.	3.35±0.95	64	3.84±1.09	64	0.382
Post.	2.19±0.87	64	2.37±0.71	64	0.064
GF	1.63±0.54	64	1.94±0.70	64	0.005*

**Table 4:** Mean±standard deviation and p-values of ant., sup., and post. joint spaces (mm) and GF roof thickness (mm) in relation to genders of edentulous patients. *Statistically significant values. Meas: measurements; n: number; GF: glenoid fossa; Ant.: anterior; Sup.: superior; post.: posterior

Joint space and GF thickness measurements	Females		Males		p-Value
	Measurements (mm)	N	Measurements (mm)	N	
Ant.	2.65±0.71	36	3.37±1.37	56	0.012*
Sup.	3.07±0.75	36	4.52±1.02	56	0.019*
Post.	1.87±0.57	36	2.26±0.72	56	0.007*
GF	1.72±0.56	36	2.01±0.71	56	0.090

## Discussion

TMJ is the sole movable joint in the cranial region, comprising the articular disc, joint capsule, joint ligaments, and the lower and upper articular surface. One factor that influences the distance between the articular surfaces and, consequently, the position of the condyle within the joint is the occlusal relationship between the mandibular and maxillar dental arches [14]. In edentulous patients, the absence of posterior occlusal contact points can affect this relationship. The condyle's position within the articular fossa may change as the remaining alveolar ridges in the maxilla and mandible come closer together [4]. Clinically, assessing condylar positioning is crucial for identifying potential risk factors for future TMJ disorders [1]. It has been proposed that in edentulous individuals, the condyle may rotate backward and upward with the anterior and posterior movement of mandible, leading to a decrease in the posterior joint space, an increase in the anterior joint space, and a forward displacement of the anterior joint space [4].

The findings of this study indicated that the condyle head was positioned more posteriorly in GF due to tooth loss in both males and females. This observation aligns with the results of Amorim et al., who reported a decrease in the posterior joint space in edentulous individuals, attributing this change to the lack of posterior tooth support [6]. Furthermore, it has been suggested that this shift may increase the risk of anterior disc displacement and subsequent temporomandibular disorders. Yanikoglu and Guldag noted that the upper joint space is relatively more minor in Kennedy Class 1 individuals compared to Kennedy Class 2 individuals, with the condyle head tending to move backward and upward as a result of reduced upper joint space due to tooth loss [5]. Similarly, Tabatabaei et al. found that while the anterior joint space of the TMJ was not significantly affected by the severity of tooth loss, the superior and posterior joint spaces were correlated with the extent of tooth loss in the posterior arches [1]. These alterations can potentially disturb the functional harmony of the TMJ and the masticatory system.

Contrary to the findings of this study, Arıkan et al. conducted a study that reported no significant difference in the posterior joint space associated with missing teeth [7]. Although they used CBCT images in their study and the measurement techniques were similar to the present study, the observed difference may have been due to the difference in sample size. In this study, G*power analysis was used to calculate the sample size, and the minimum sample size was calculated as 44. However, 24 samples were taken for both groups in the study by Arıkan et al. [7].

The bone above the articular fossa usually thickens in response when bone resorption occurs in the condyle. This thickened bone structure is adapted to support the increased stress on the TMJ caused by condylar bone changes. However, most of the toothless individuals are elderly and have reduced tissue regeneration capacity and metabolic function, which may prevent adequate compensation of the bone over the articular fossa. As a result, abnormal alterations in the TMJ bone structure can improve joint stress and lead to functional changes in the TMJ. Significant functional changes in edentulous patients can result in the mandibular fossa adapting as part of the overall functional system and becoming a unit of reconstruction [4]. In our study, we classified the patients according to their edentulous status, not according to their age.

In a comparative CBCT study of 100 patients by Rosado et al., edentulous individuals exhibited a higher incidence of bone changes in the condyle, including osteophytes, resorption, and sclerosis [9]. In addition, these patients showed a thinner glenoid fossa roof and a steeper lateral slope of the glenoid fossa compared to edentulous individuals. The edentulous individuals had a lower GF roof thickness compared to the toothed group, possibly due to orthopedic instability. Also, since the toothed individuals had more significant occlusal stability than the edentulous patients, they may have better distributed masticatory forces. Veettil et al. measured the GF roof thickness of 120 TMJ in toothed, partially edentulous, and completely edentulous patients [8]. They reported that the GF roof thickness had no relationship with the edentulous status of the patients and that there were no age- or gender-related differences. In this study, no significant difference was found in the thickness of the GF roof between edentulous and dentulous patients.

Patterns of bone loss may differ between the genders. It is accepted that significant bone loss begins at menopause in females due to sex steroid deficiency, whereas in males it begins later in life due to age-related factors [15]. In their study evaluating occlusal stability and degenerative changes in the temporomandibular joints, Magnusson et al. reported that loss of occlusal support was not a predisposing factor for degenerative changes in males, but they found a correlation, especially in older females [16]. In the study conducted by Yun et al., it was reported that bone thickness measurements were higher in males due to skeletal differences [17]. This finding explains the higher GF values ​​in our study. In the same study, higher values ​​were obtained in males in anterior, superior, and posterior joint space measurements, similar to our study, but no statistically significant difference was found [17]. In the current study, joint space values ​​were measured higher in males; however, statistically significant differences were found between the genders in the anterior joint space in dentate individuals and in all joint spaces in edentulous individuals. This finding suggests that both edentulism and age-related changes may serve as significant determinants of temporomandibular joint space dimensions.

In addition to the facial profile, both environmental and genetic factors can influence an individual’s stomatognathic system and subsequently affect the bony morphology of the mandible. Degenerative changes can occur in the TMJ when masticatory function is altered or when a significant force disrupts the joint structure. Loss of occlusal support, which can disrupt the balance in the masticatory system, is one of the potential factors that can accelerate the deterioration of the TMJ [18].

Considering the complexity of TMJ components, numerous studies have investigated the morphologic parameters of the TMJ using various imaging techniques. Traditional X-ray lateral cephalometric and panoramic radiography [3,4], computed tomography [19], CBCT [1,9,11,12], magnetic resonance imaging [20], and ultrasound are the imaging modalities used to investigate TMJ morphology [21]. The use of CBCT has become an increasingly important resource in diagnosing TMJ disorders, especially in evaluating structural changes in the mandibular condyles. CBCT has been shown to obtain images with high resolution, proving clear visualization of the bony tissues of the TMJ, and has been shown to reduce radiation and cost significantly compared to CT [11,22]. The CBCT imaging system has allowed accurate visualization of the important buccolingual dimensions as well as the vertical dimensions in the mandible in real size [23]. As a newly developed imaging technique in dentistry, CBCT is likely to be the most effective method for evaluating the bone morphology of the TMJ joints [24]. Therefore, CBCT was used in this study.

CBCT, or dental volumetric CT, utilizes a cone-shaped X-ray beam as opposed to the parallel fan beam used in spiral CT. The scanning process involves the tube-detector system rotating 360 degrees around the patient's head while the beam angle remains constant. This rotation generates the initial raw data, which is presented as a lateral tomogram and is then used for primary reconstruction. The layer thickness options for reconstruction are 3.0 mm, 1.0 mm, and 0.3 mm, and the clinician determines the reconstruction angles. These primary images are suitable for secondary reconstructions and 3D reconstructions across all planes. The orientation of the reconstruction is adjusted according to the angle of the condyle to visualize the position of condyle accurately. Numerous research indicate that higher diagnostic quality images are achieved when reconstructed images are aligned perpendicular or parallel to the condyle long axis [25]. The CBCT images in this study using the same criteria reached diagnostic quality.

Limitations

In our study, there is a lack of information about important factors such as patients' lifestyle habits, body mass indexes, and nutritional habits. Possible differences between patients in these unknown variables could have potentially influenced our findings. In particular, factors such as lifestyle habits (e.g., bruxism or jaw-shifting habits) and nutritional habits (e.g., behaviors that put excessive stress on the joint, such as constantly chewing hard foods or breaking shelled foods with the teeth) may play an important role in joint health.

Additionally, increasing the amount of patient data may allow other critical variables, such as the duration of edentulism, to be included in the study or age-related changes should be taken into account. This would contribute to a more detailed evaluation of the findings and enhance the overall validity of the study. However, the aforementioned limitation in the existing data set restricted the possibility of conducting such an analysis and reduced the precision of the findings.

## Conclusions

This study revealed that there was no significant relationship between tooth loss and changes in the upper joint space of the TMJ. However, this situation is directly related to both posterior and anterior joint spaces, and this relationship manifests itself as a decrease in the posterior joint space and an increase in the anterior joint space with edentulism. This finding suggests that the mandibular condyle head tends to move posteriorly in cases of edentulism. On the other hand, GF roof thickness was not associated with edentulism. Nevertheless, based on this information, it can be concluded that significant functional changes, such as those observed in edentulous patients, lead to a remodeling of the fossa as part of the overall functional adaptation. Rapidly restoring missing teeth is essential to maintain the TMJ and the stomatognathic system balance.
